# The Sensation of Thirst

**Published:** 1882-05

**Authors:** 


### THE SENSATION OF THIRST.

Professor Nothnagel contributed a very interesting and instructive article to a recent number of Virchow's *Archiv*, upon which the London *Lancet* comments, and from which we extract the salient points. It is well known that the sensation of thirst expresses an insufficiency in the amount of water in the organism. But why is thirst referred to the mouth and throat? The scanty experimental researches on the subject teach but little. He relates a remarkable case, which throws some light on the question. A man was kicked in the abdomen by a horse, and knocked down, striking the occiput against the hard ground. He was not stunned, but could not walk, on account of the severe pain in the abdomen and back of the head and neck. Previously well, immediately after the accident he began to feel extreme thirst, and in the next three hours drank five pints of liquid. No injury to bone could be detected, temperature was normal, and pupils small. The local pain soon passed away, but the thirst continued. The thirst was unabated during the three weeks the patient was under observation. It was uninfluenced by treatment, and became more intense every day about noon. Nothing but large quantities of water relieved it, the amount taken daily varying from one to four gallons. The quantity of urine secreted daily was about three-fourths the amount of water taken, and its specific gravity varied from 1.002 to 1.010. The skin was moist. Nothnagel believed that the thirst must have been due to a central effect of the blow on the occiput. The probabilities, he thought, are that the thirst centre is located in the pons or medulla; though this is only speculation, since there were no symptoms sufficiently distinctive to accurately locate it.—*Medical and Surgical Reporter.*

